# Three-Dimensional Reproducibility of the Soft Tissue Landmarks Taken by Structured-Light Facial Scanner in Accordance with the Head Position Change

**DOI:** 10.3390/healthcare9040428

**Published:** 2021-04-07

**Authors:** Seung-Hoon Oh, Jung-Soo Park, Jae-Jun Ryu, In-Seok Song, Seok-Ki Jung

**Affiliations:** 1 Department of Orthodontics, Graduate School of Clinical Dentistry, Korea University, Seoul 02841, Korea; oshoong@naver.com; 2 Department of Periodontology, Korea University Anam Hospital, Seoul 02841, Korea; freeme82@korea.ac.kr; 3 Department of Prosthodontics, Korea University Anam Hospital, Seoul 02841, Korea; kopros@korea.ac.kr; 4 Department of Oral and Maxillofacial Surgery, Korea University Anam Hospital, Seoul 02841, Korea; 5 Department of Orthodontics, Korea University Guro Hospital, Seoul 08308, Korea

**Keywords:** structured-light facial scanner, soft tissue landmarks, head position, reproducibility

## Abstract

The aim of this study was to evaluate the three-dimensional reproducibility of the structured-light facial scanner according to the head position change. A mannequin head was used and angle of the mannequin’s axis-orbital plane to the true horizontal plane was adjusted to +10, +5, 0, −5, and −10°. Facial scanning was conducted 30 times, respectively, and 150 3D images were obtained. Reoriented landmarks of each group were compared and analyzed. Reproducibility decreased as the distance from the facial center increased. Additionally, the landmarks below showed lower reproducibility and higher dispersion than landmarks above. These differences occurred mainly in the anteroposterior direction as opposed to other directions. Positive inclination of the head position showed superior reproducibility compared to a negative inclination. This study showed that reproducibility of a structured-light scanner could be varied depending on the head position. Inaccuracies of landmarks in the anteroposterior direction are greater than in other directions. This means that evaluations of the profile using a structured-light scanner should be made carefully. Therefore, the proper head position should be set to ensure the accuracy of the image.

## 1. Introduction

The paradigm of orthodontics has shifted to the soft tissue [1]. Though orthodontic movement is performed in the hard tissue, it is important to assess the soft tissue accurately. This is because the soft tissue profile is the part of interest for patients. Various diagnostic data are used for evaluation of the soft tissue. The most typical diagnostic method is taking clinical photos which show lateral and frontal views. Lateral photos are useful in soft tissue prediction for visual treatment objectives (VTOs) and surgical treatment objectives (STOs) after orthodontic treatment. However, it has limitations in diagnosis and treatment planning since it only provides two-dimensional information [2]. In addition, patients are interested in the change in frontal face according to the change in the lateral profile. In order to overcome these points, various 3D analysis methods exist such as photogrammetry, laser scans, structured-light facial scans, video imaging, computed tomography (CT), and magnetic resonance imaging (MRI) [3,4,5,6,7]. Video imaging has the advantage of recording the actual dynamic movement of the face. However, it has the disadvantage of difficulty in acquiring accurate measurements. CT has an advantage in that it provides information of the hard tissue and the soft tissue [8]. However, it has the weakness of unnecessary exposure to radiation.

3D facial scanners are less invasive, cheaper and faster than X-ray devices. Therefore, clinicians can use 3D facial scanners for facial measurements. These 3D devices include laser scanners [9,10], stereophotogrammetry [11,12] and structured-light systems [13,14]. Stereophotogrammetry is used to measure the three-dimensional location of landmarks on the surface of the face. Laser scanners accurately capture 3D shapes in millions of points based on triangulation. Since the sensor is at a known distance from the laser source, it is possible to perform accurate point measurements by calculating the angle of reflection of the laser light. The advantage of laser scanning is its resolution and accuracy. However, it is important to note that the properties of the surface to be scanned affect the scanning process.

Recently, facial scanning using structured light has been rapidly developed. It obtains a 3D image of face by matching up the front and lateral images of patient’s face. This method has the advantage of simplicity without radiation exposure, which can provide comfort to both patients and practitioners. However, it may have a weakness in terms of distortion since facial images are made by superimposing only three pictures. Actually, a structured-light facial scanner reconstructs the 3D image by shooting the visible structured light to the object and recognizes the pattern image reflected from the surface. Thus, accurate and precise scanning must be followed to reduce the distortion. There were many previous studies evaluating the reproducibility of the 3D facial scanning method [15,16,17,18,19]. However, most research papers were focused on the laser scanning method, and only a few were examined based on the structured-light facial scanning method [20,21]. Moreover, three-dimensional reproducibility of landmarks followed by change in the head position has not been discussed. When taking CT images, head position change cannot affect the result of the image. However, structured-light facial scanning can be affected by change in the head position. Actually, if the head position is changed in the upper or lower direction, structured-light scan images are likely to lead to distortion of particular landmarks. The shade according to the curvature of the face could affect the imaging process and distortion in 3D image. If this kind of error occurs, comparison between two 3D images might not have any meaning.

The aim of this study was to evaluate three-dimensional reproducibility of the structured-light facial scanner according to the head position change. This study aims to present some guidelines for practitioners about structured-light scanning.

## 2. Materials and Methods

**Figure 1:**
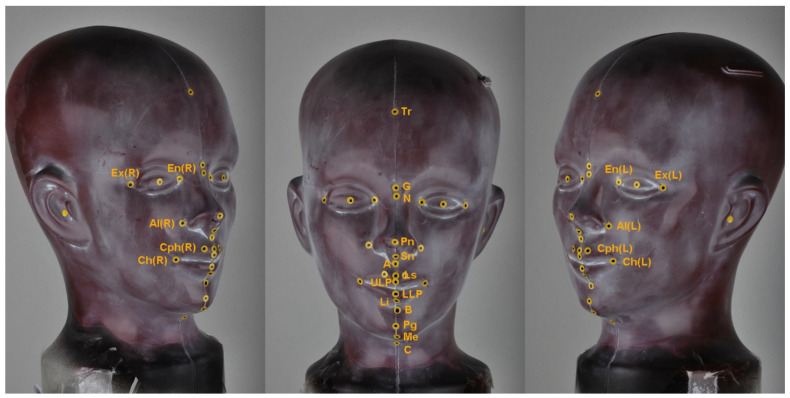
Soft tissue landmarks used in this study: Ex (R), exocanthion on the right side; Ex (L), exocantion on the left side; En (R), endocanthion on the right side; En (L), endocanthion on the left side; Tr, trichion; G, glabella; N, nasion; Pn, pronasale; Sn, subnasale; Al (R), alare on the right side; Al (L), alare on the left side; A, A point; ULP, upper lip point; LLP, lower lip point; B, B point; Pg, pogonion; Gn, gnathion; Me, menton; C, cervical point; Ls, labrale superius; Li, labrale inferius; Ch (R), cheilion on the right side; Ch (L), cheilion on the left side; Cph (R), crista philtre on the right side; Cph (L), crista philtre on the left.

A mannequin head was prepared to evaluate three-dimensional reproducibility of the soft tissue landmarks according to the change in head position. Soft tissue landmarks of the mannequin head were marked with circular stickers which had point at their centers (Figure 1).

**Figure 2:**
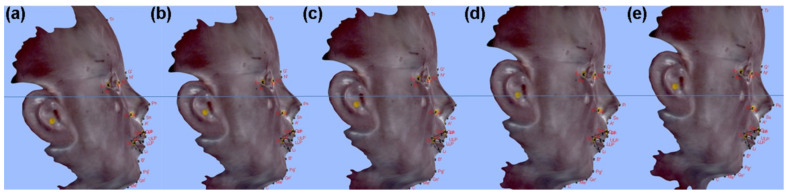
Groups used in this study: (**a**) Group D+10°; (**b**) Group D+5°; (**c**) Group D−0°; (**d**) Group D−5°; (**e**) Group D−10°.

Facial scanning was carried out by a structured-light scanner (Morpheus 3D, Morpheus Co., Seoul, Korea) at the same place with constant lighting as instructed by the manufacturer. Angle of the mannequin’s axis-orbital plane to the true horizontal plane was adjusted to the +10, +5, 0, −5, −10° using an angle adjustment device. Despite the angle adjustment, the distance between the light source and pronasale (Pn) was maintained. Each group was named sequentially as Group D+10°, Group D+5°, Group D−0°, Group D−5°, and Group D−10° (Figure 2).

Facial scanning was conducted 30 times for each group, respectively, and 150 3D images was obtained. Every scanning process was performed by a single investigator. Through the imaging process, coordinates of every soft tissue landmark were obtained. The vertical axis was set as *z*-axis, the anteroposterior axis was set as the *x*-axis, and the transverse axis was set as the *y*-axis. Coordinates of landmarks in each group were reoriented using MATLAB (ver 8, MathWorks Inc., Natick, MA, USA).

Reproducibility was evaluated by comparing landmarks of each group. In the 3D coordinates, a circle with a center of the median value and a radius of the interquartile range (IQR) was drawn for every landmark of each group. The IQR is a measure of variability, based on dividing a data set into quartiles. The IQR is the first quartile subtracted from the third quartile. In order to evaluate this, frontal and lateral views were illustrated using language R-program (http://www.r-project.org/, accessed on 27 December 2020. R Development Core Team. 2013) [22]. Three-dimensional distribution of the median value was analyzed for every landmark of each group in comparison with Group D−0°. In addition, an analysis of variance (ANOVA) test and Tukey HSD post hoc test were performed to determine the degree of match in landmarks between each group and Group D−0°.

## 3. Results

**Figure 3:**
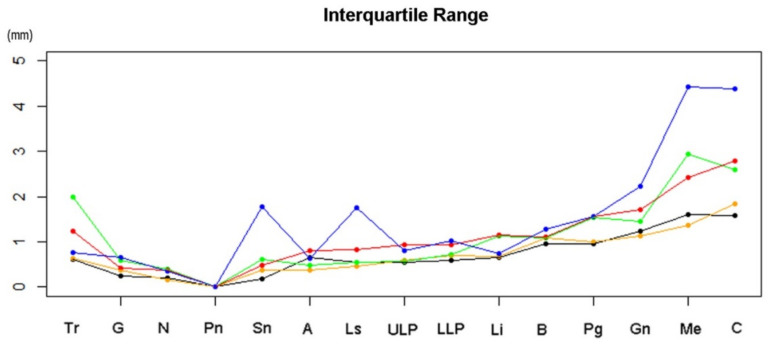
Interquartile Range (IQR) distribution: D+10° (**red**), D+5° (**orange**), D−0° (**black**), D−5° (**green**), and D−10° (**blue**).

Median value and IQR of coordinates of landmarks in each group were shown in Table 1. IQR increased at the landmarks that were further from the facial center. Landmarks below showed higher dispersion than landmarks above (Figure 3).

Group D−0° showed the smallest IQR, which increased in the order of Group D+5°, D−5°, D+10°, and D−10°. Differences in median value between each group and Group D−0° were exhibited by anteroposterior, transverse, vertical, and total distances in a three-dimensional space. The difference was largest in the anteroposterior direction. The next was in the vertical direction, and the minimum was in the transverse direction (Table 1).

**Figure 4:**
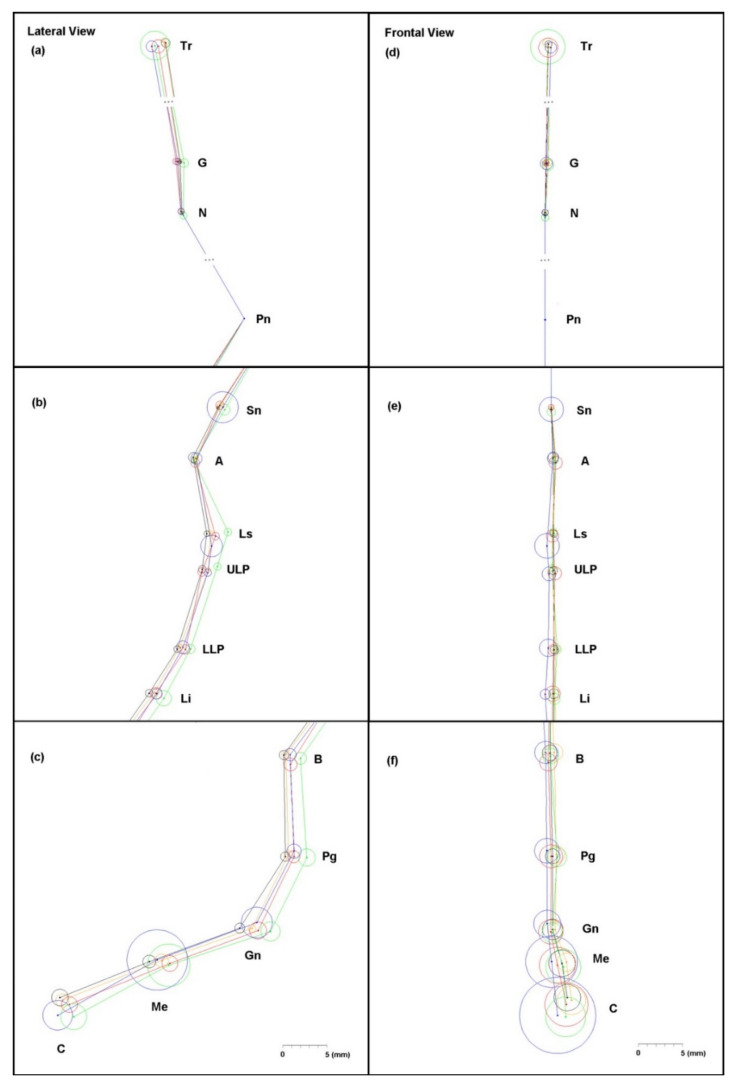
Lateral (**a**,**b**,**c**) and frontal (**d**,**e**,**f**) views of distributions of the landmarks: D+10° (**red**), D+5° (**orange**), D−0° (**black**), D−5° (**green**), and D−10° (**blue**).

In addition, differences in the median increased as the distance from the facial center increased, particularly at the landmarks below. In contrast with other landmarks, the vertical distance had the most impact on the total distance in the C point. In order to investigate the three-dimensional distribution of landmarks, lateral and frontal views of landmarks are illustrated in Figure 4. A circle with a center at the median value and a radius of the IQR was drawn for every landmark of each group. In this figure, difference of median value between groups was smaller in the frontal view than the lateral view. As noted earlier, this difference was mainly due to anteroposterior differences except for the case of the C point, which has major difference in the vertical direction. Similarly with the lateral view, the frontal view also showed lower reproducibility and higher dispersion at the landmarks below. Both lateral and frontal views showed the largest variances in Group D−10°.

**Figure 5:**
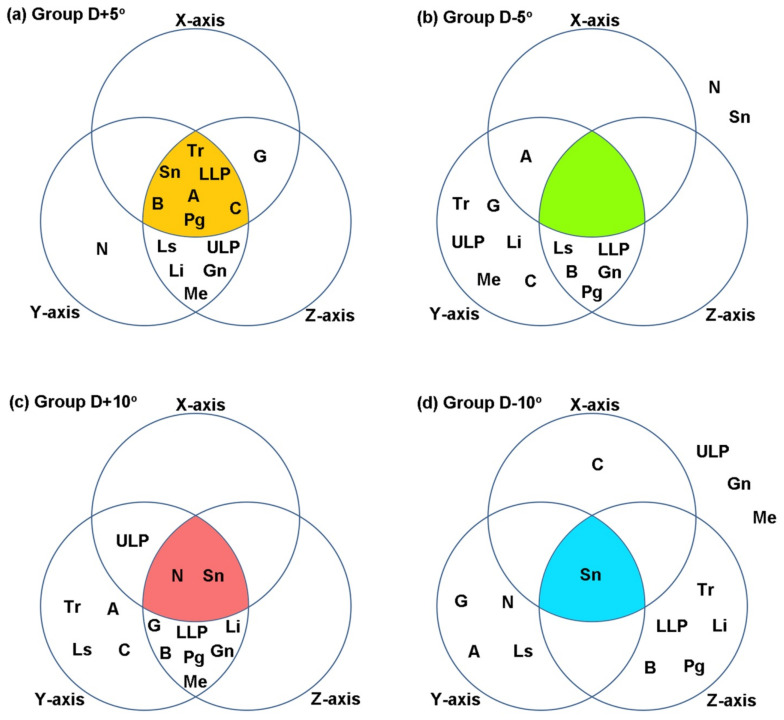
Diagrams for landmarks having no difference with Group D−0° in each axis: (**a**) Group D+5°; (**b**) Group D−5°; (**c**) Group D+10°; (**d**) Group D−10°.

To compare the differences of median value between each group and Group D−0°, one-way ANOVA was performed with respect to each axis in the three-dimensional coordinates, and Tukey HSD test was performed as a post hoc test. Diagrams for each group were drawn (Figure 5). Each circle contained landmarks which were not different with those of Group D−0° in each axis. The elements in the intersection of three circles were the landmarks which were not different to those of Group D−0° on every axis. Group D+5° showed no difference with Group D−0° in seven landmarks, which is the largest. There were two landmarks for Group D+10°, one for Group D−10°, and none for Group D−5°.

## 4. Discussion

The lower face is the area most affected by orthodontic treatment or orthognathic surgery [23,24,25]. Soft tissue changes in this area would be the most concerns to both patients and practitioners. Using conventional two-dimensional diagnostic methods, it has been difficult to show effect of orthodontic treatment three-dimensionally. On the other hand, 3D facial scanners enable simulation of soft tissue change followed by hard tissue change and can give us realistic information [2].

Using facial scanners to integrate three photos into a three-dimensional image is easy and simple. However, this leads to the possibility of distortion. As shown in this study, accuracy and reproducibility became lower in the landmarks far from the facial center. This is important because it can affect the accuracy in evaluating the lower face. In particular, landmarks such as Gn, Me, and C point which are located around the chin and neck areas, showed wider distributions. Similarly, in a previous study using a laser scanner, errors mostly occurred in landmarks around the chin area [26]. The reason for this could be that the chin and neck areas have poor visibility. Empty space in the 3D image was filled up using a calculation of the program, which could affect the result.

According to this study, changes in three-dimensional position of landmarks take place mainly in the anteroposterior direction. The cause for this could be the process of matching three images (frontal, left and right 45°) into a 3D image. This process could lead to smaller distortion in the vertical and transverse directions. This is because the vertical information was the actual size in the matching process while anteroposterior information should be calculated by the program. For a similar reason, distortion in the transverse direction was small as the frontal image was already given. Therefore, distortion in anteroposterior direction was major distortion in the 3D image. However, at point C, the distortion in the vertical direction was greater. The reason seems to be due to the undercut area around the chin.

Moreover, negative inclination groups such as Group D−5° and D−10° tended to show lower reproducibility than positive inclination groups. Structured-light scanner shoots multislit beams into the face and integrates three-dimensional image by recognizing reflecting patterns [2]. Much more shadow could be formed in the lower area for the negative inclination groups than the positive inclination groups. Therefore, this can lead more errors. In this study, Group D−10° showed the greatest dispersion.

A limitation of this study is that three images (frontal, left, right) were not taken simultaneously—they were taken separately three times by one scanner. For this reason, inaccuracy could be increased when integration had been carried out. To overcome this disadvantage, the manufacturer recommends using three scanners at the same time. However, the cost could be the problem. In addition, a mannequin head was managed in static position, and scanning was carried out repeatedly without change in position. However, it can be different for a human face. Moreover, its characteristics such as surface texture, color, and reflectivity can be also different with those of mannequins. Further research is required on real human faces. As we tried to verify the difference in reproducibility of the landmarks according to changes in fine angles, it was difficult to experiment with real people. The model of the mannequin was more suitable to reproduce the exact angle change. However, in-depth research is necessary because there may be differences from the results of mannequins in the actual human body.

In summary, structured-light scanners are easy and simple to use, but also have a relatively low accuracy in the linear measurements than laser scanner or CT. This implies that a suitable choice is required for adequate usage. In addition, it is important to know that inaccuracies of landmarks in the anteroposterior direction are greater than in other directions. This means that evaluation of the profile using a structured-light scanner should be careful. However, asymmetric analysis in structured-light scanners can be more reliable as errors in frontal view are small [27,28]. Moreover, inclination of the head position in the sagittal plane can change the reproducibility. In particular, negative inclination of the head position can cause worse results than positive inclination. Therefore, proper head position should be set to take an accurate image. Several methods can be proposed for this. The first one that can be considered is the natural head position. Patients can position their heads in the most natural position by looking in the mirror. Another method is to use an FH plane, which allows the operator to mark the plane and attach a level to the position. A reproducible position is desirable no matter which method is used.

## Figures and Tables

**Table 1:** Median coordinates and interquartile range (IQR) of each group (mm).

	Group D−0°				Group D+5°				Group D−5°				Group D+10°				Group D−10°			
	Median			IQR	Median			IQR	Median			IQR	Median			IQR	Median			IQR
	x	y	z		x	y	z		x	y	z		x	y	z		x	y	z	
**Tr**	−25.59	1.40	88.60	0.61	−25.69	1.32	88.73	0.64	−26.82	1.32	88.30	1.99	−26.43	1.38	88.20	1.24	−27.18	1.68	88.21	0.76
**G**	−17.19	0.13	35.28	0.24	−17.31	0.35	35.36	0.37	−16.75	0.34	35.13	0.58	−17.66	0.11	35.32	0.43	−17.46	0.19	35.29	0.64
**N**	−16.96	0.00	29.38	0.19	−17.10	0.00	29.62	0.16	−16.79	0.00	29.08	0.40	−17.07	0.00	29.57	0.36	−17.07	0.00	29.57	0.34
**Sn**	−7.95	0.00	−12.26	0.18	−7.67	0.00	−12.06	0.38	−7.21	0.00	−12.49	0.61	−7.73	0.00	−11.98	0.47	−7.42	0.00	−12.21	1.78
**A**	−10.55	0.23	−17.53	0.65	−10.29	0.40	−17.62	0.38	−10.47	0.40	−17.89	0.49	−10.32	0.50	−18.17	0.80	−10.19	0.16	−17.67	0.64
**Ls**	−9.01	0.25	−26.27	0.56	−8.60	0.12	−26.26	0.45	−6.61	0.21	−26.08	0.55	−8.02	0.10	−26.56	0.82	−8.47	−0.52	−27.68	1.76
**ULP**	−9.53	0.21	−30.30	0.55	−8.83	0.09	−30.40	0.59	−7.82	0.05	−30.00	0.56	−9.56	0.37	−30.60	0.93	−8.91	−0.30	−30.68	0.80
**LLP**	−12.40	0.31	−39.41	0.58	−12.12	0.47	−39.22	0.70	−10.89	0.65	−39.39	0.73	−11.48	0.22	−39.43	0.92	−11.76	−0.37	−39.19	1.03
**Li**	−15.58	0.24	−44.42	0.66	−14.99	0.33	−44.37	0.68	−13.91	0.31	−45.01	1.12	−14.79	0.12	−44.43	1.15	−14.69	−0.71	−44.50	0.73
**B**	−20.91	0.24	−51.92	0.95	−20.69	0.68	−51.86	1.08	−19.00	0.29	−52.29	1.09	−20.14	0.04	−53.00	1.10	−20.20	−0.28	−51.88	1.28
**Pg**	−20.75	0.57	−63.45	0.95	−20.40	0.99	−63.23	1.01	−18.30	1.07	−63.54	1.53	−19.77	0.39	−63.45	1.55	−19.76	−0.09	−62.81	1.57
**Gn**	−25.92	0.58	−71.56	1.24	−24.51	0.85	−71.59	1.13	−22.40	0.52	−71.93	1.46	−23.83	0.36	−71.83	1.72	−23.97	−0.04	−70.91	2.23
**Me**	−36.24	1.65	−75.39	1.61	−34.83	2.16	−75.27	1.37	−34.03	1.80	−75.81	2.94	−33.90	1.12	−75.60	2.42	−35.34	0.46	−75.20	4.44
**C**	−46.41	2.27	−79.49	1.59	−46.12	2.75	−79.82	1.84	−44.84	2.09	−81.69	2.60	−45.32	2.18	−80.31	2.80	−46.66	1.17	−81.53	4.39

## Data Availability

The data underlying this study will be available on reasonable request to the corresponding author.
